# Supplementary figures and images for: Modulation of nucleotide metabolism by picornaviruses
Source: PLoS Pathog. 2024 Mar 8;20(3):e1012036. doi: 10.1371/journal.ppat.1012036 (PMC10923435; doi:10.1371/journal.ppat.1012036)

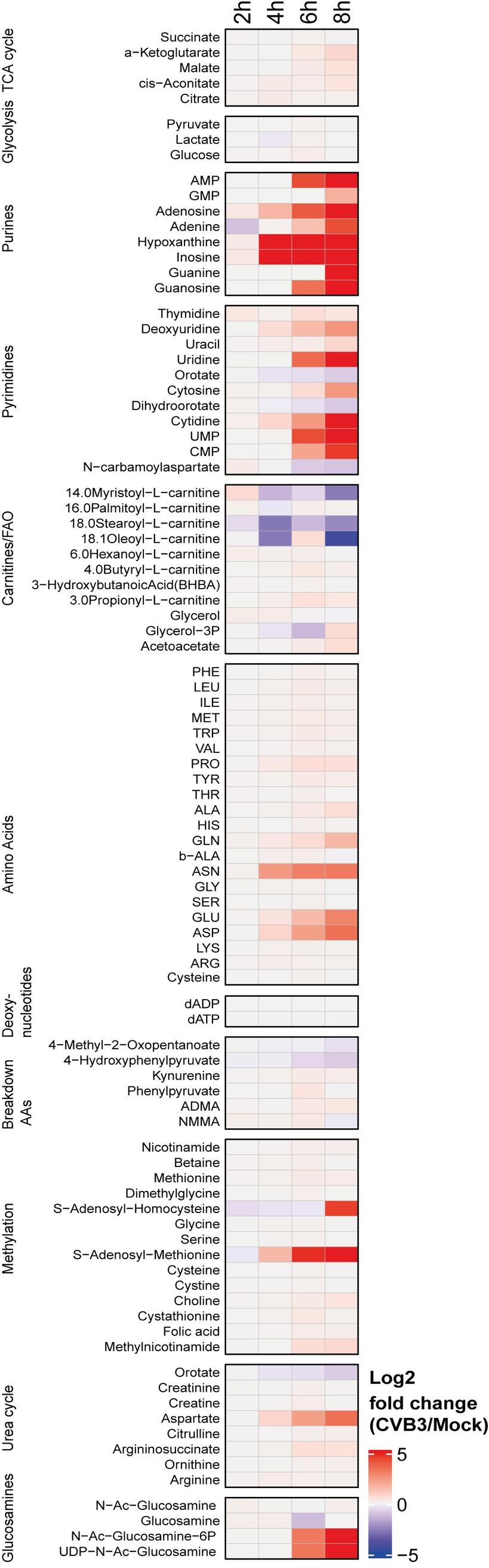

Supplement: S1 Fig — 13C-glucose isotope tracing study in mock- and CVB3-infected HeLa R19 cells (three replicates; one experiment; MOI 5). Cells were infected, lysed at 2,4,6 or 8 hpi and measured by LC-MS to identify metabolites and quantify the different isotopologues. The different isotopologues are not distinguished in this Figure. Heatmap showing log2 fold changes of extracellular metabolites during CVB3 infection using. (TIF) [file ppat.1012036.s001.tif]

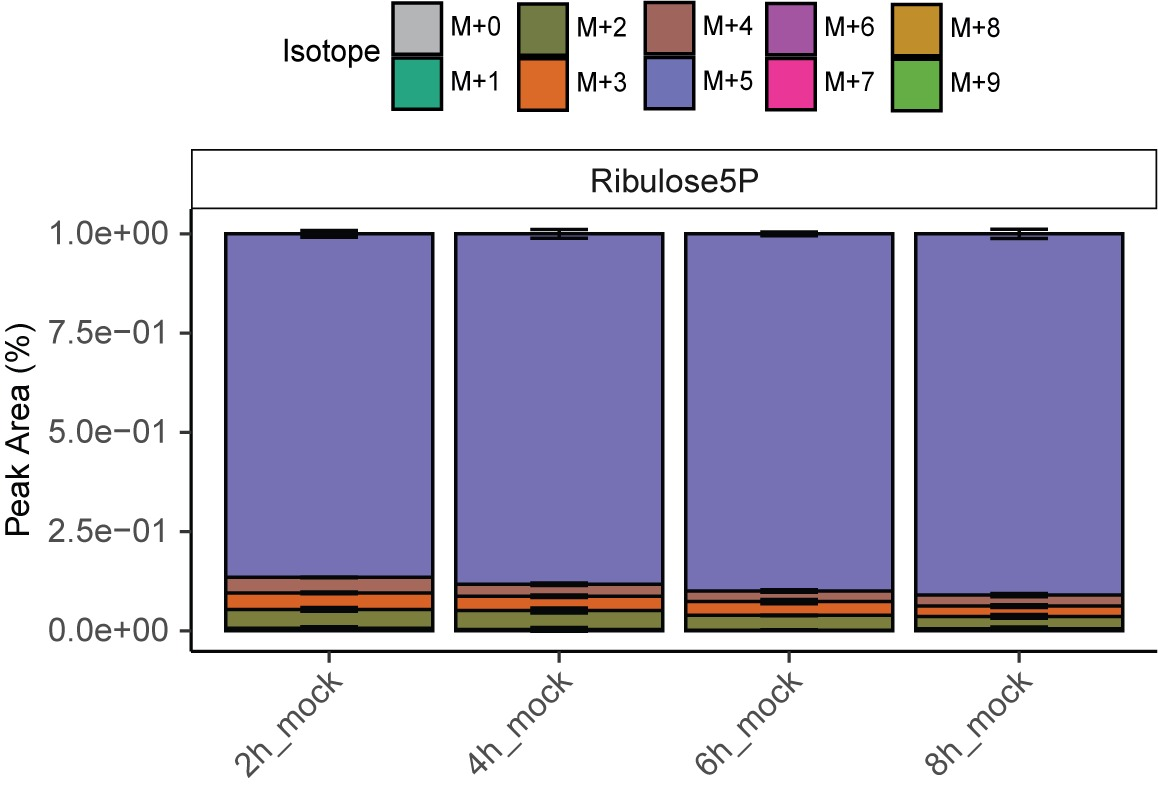

Supplement: S2 Fig — 13C-glucose isotope tracing study in mock HeLa R19 cells (three replicates; one experiment; multiplicity of infection (MOI) = 5). Cells were lysed at 2,4,6 or 8 hpi and measured by LC-MS to identify metabolites and quantify the different isotopologues. Ribulose-5-Phosphate is depicted. (TIF) [file ppat.1012036.s002.tif]

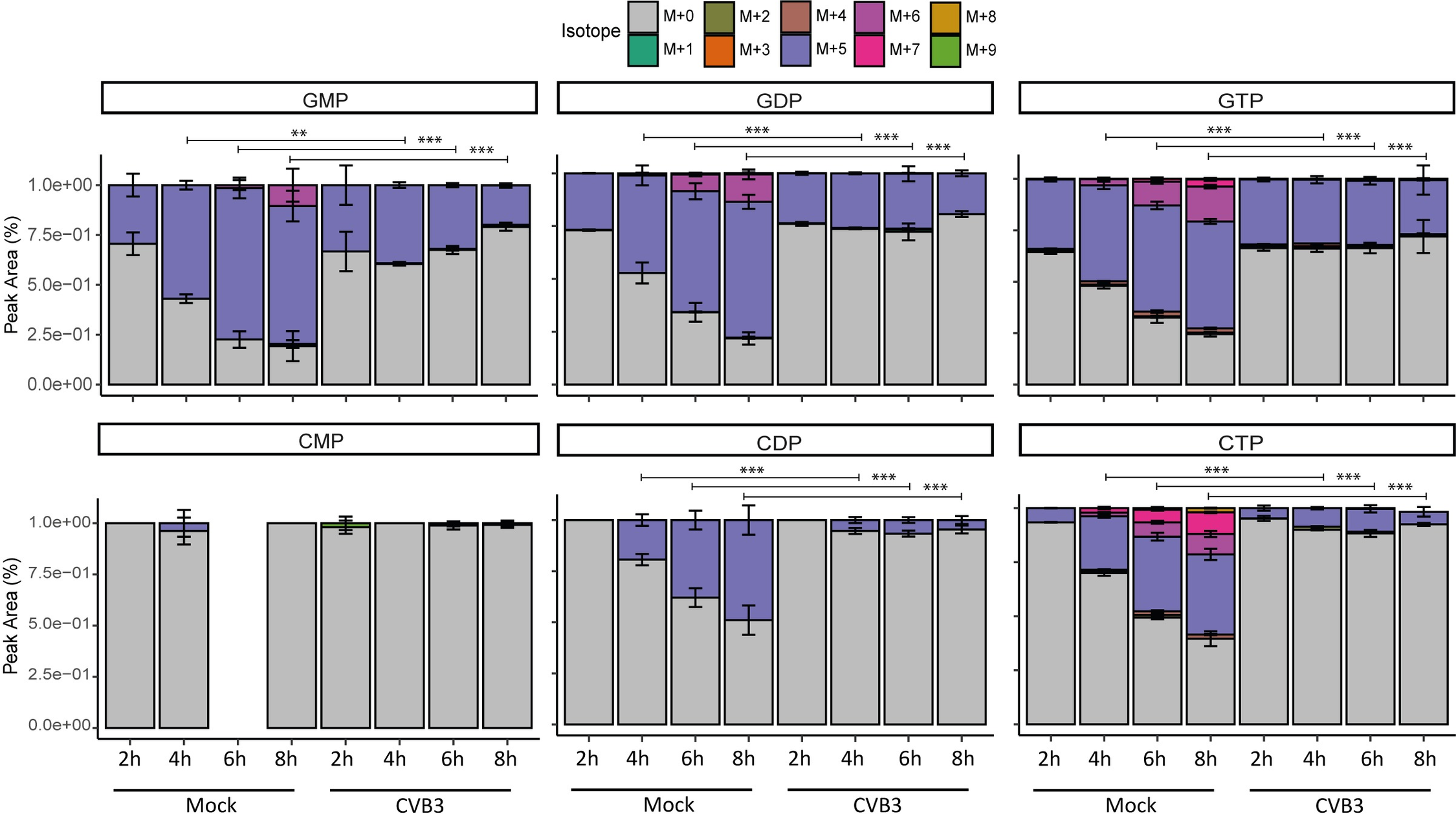

Supplement: S3 Fig — 13C-glucose isotope tracing study in mock- and CVB3-infected HeLa R19 cells (three replicates; one experiment; multiplicity of infection (MOI) = 5). Cells were infected, lysed at 2,4,6 or 8 hpi and measured by LC-MS to identify metabolites and quantify the different isotopologues. In one replicate of 6h mock, CMP was undetectable leading to inaccurate fraction calculations. Therefore, the 6h mock was omitted in the CMP figure. The p-values were calculated using linear mixed effect models with an interaction of time and treatment and a random effect of replicate. For CMP, a normal distribution of the residuals could not be assumed and therefore a non-parametric linear mixed effect model with an interaction of time and treatment and a random effect of replicate was performed. Afterwards, a contrast analysis was done to calculate the p-values between specific groups. For this analysis, the fractions of all labels were added together and tested whether the total amount of labeling differed between mock and CVB3 infection. *p < 0.05, **p < 0.01, ***p < 0.001. (TIF) [file ppat.1012036.s003.tif]

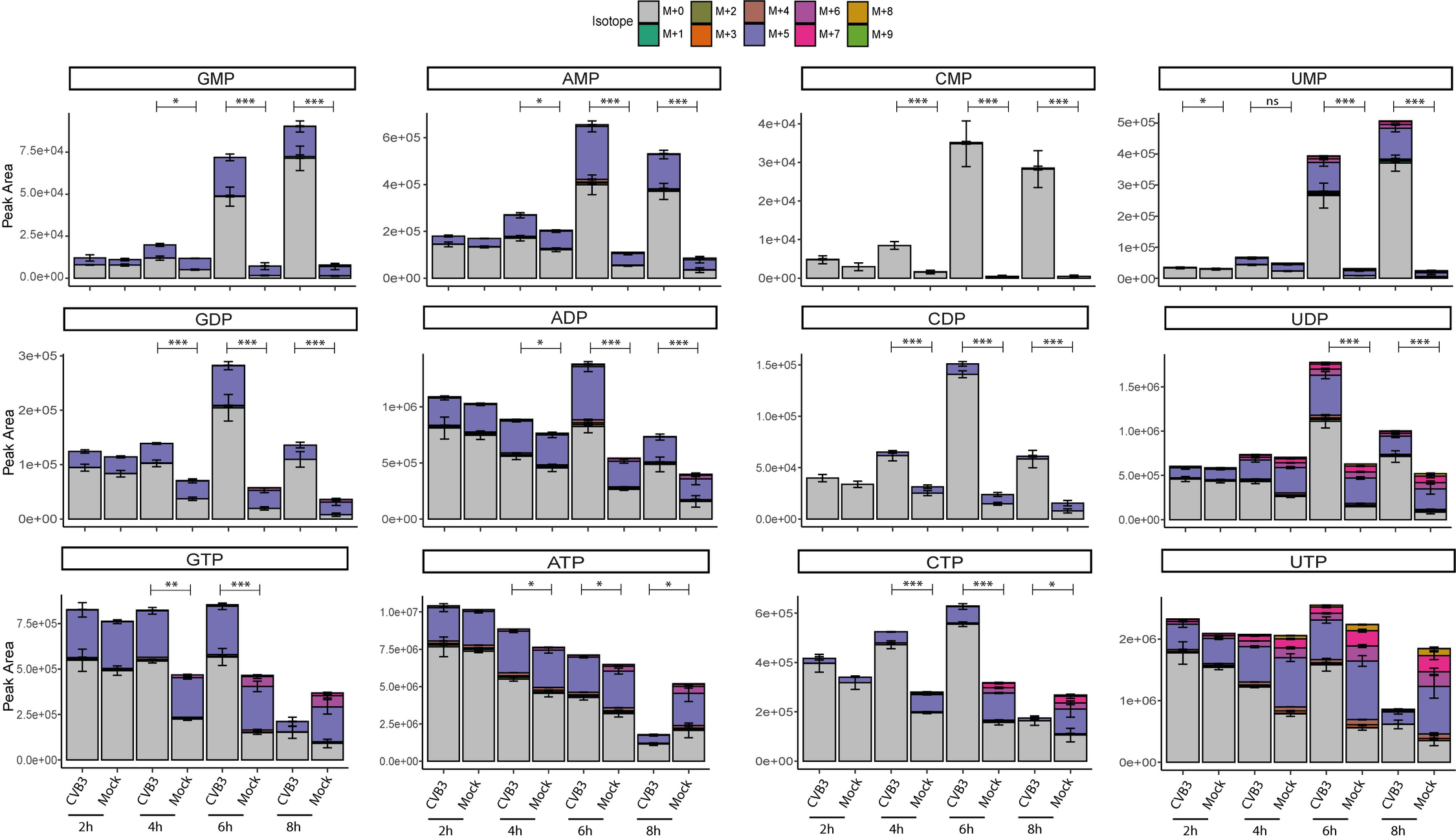

Supplement: S4 Fig — Metabolomics study of mock and CVB3 infected HeLa R19 cells (three replicates; one experiment; MOI 5). The cells were infected, lysed at 2, 4, 6 or 8 hpi and measured by LC-MS to identify metabolites. The absolute levels of nucleotide mono-, di- and triphosphates are shown. The p-values of the absolute metabolite levels were calculated using linear mixed effect models with an interaction of time and treatment and a random effect of replicate. A rank transformation on the data was performed to ensure a normal distribution of the residuals. Afterwards, a contrast analysis was done to calculate the p-values between specific groups. *p < 0.05, **p < 0.01, ***p < 0.001. (TIF) [file ppat.1012036.s004.tif]

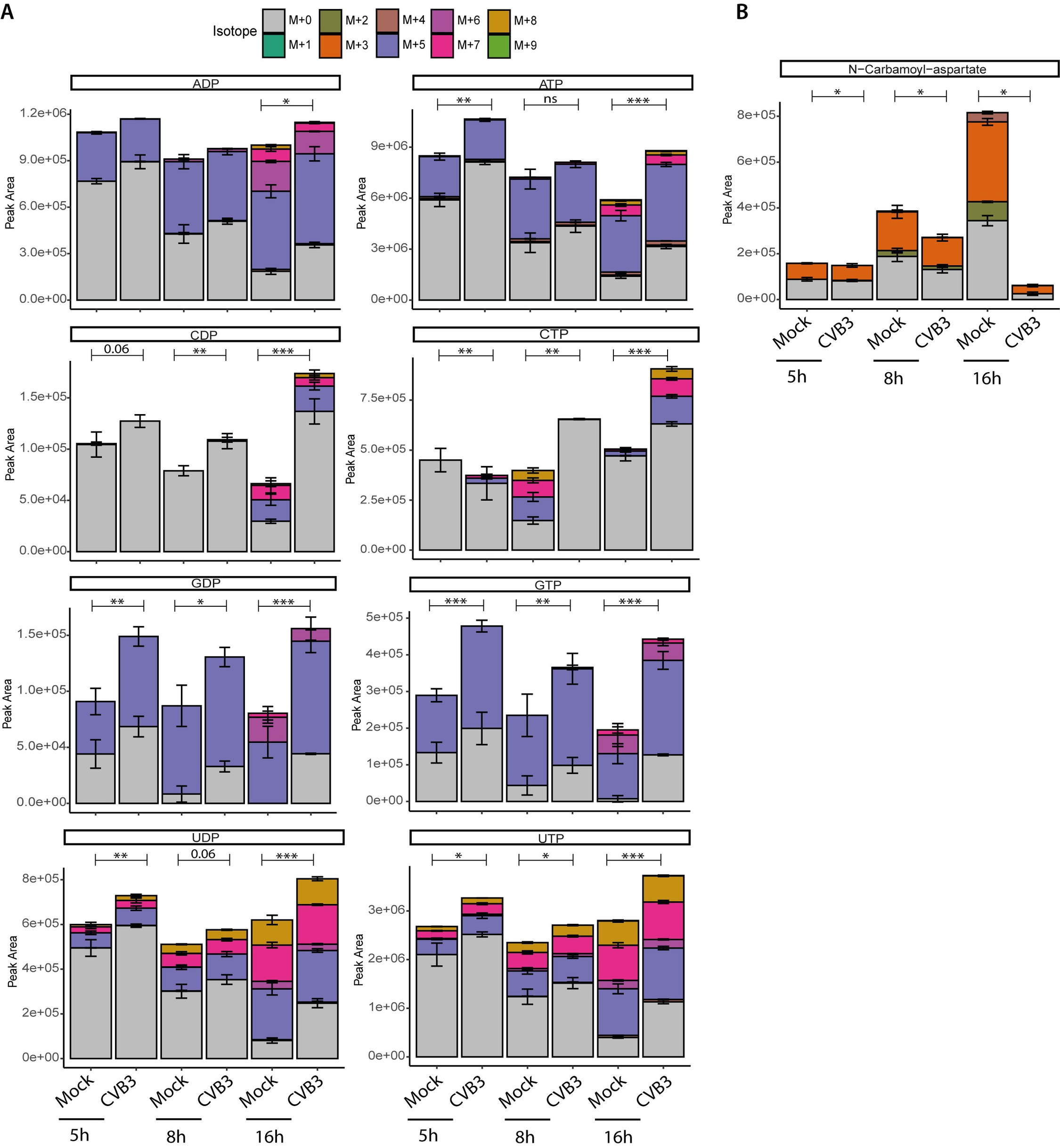

Supplement: S5 Fig — 13C glucose isotope tracing study of mock and CVB3 infected Huh7 R19 cells (three replicates; one experiment; MOI 5). The cells were infected, lysed at 5,8 or 16 hpi and measured by LC-MS to identify metabolites. One sample was removed from analysis (one replicate of 5 hpi CVB3), because of a technical defect. A) The relative contributions of the different labelings are shown for eight representative nucleotides: ADP, ATP, UDP, UTP, CDP, CTP, GDP, GTP. B) The absolute level of N-carbanoyl-aspartate in Mock and CVB3 infected samples over time. The p-values of the absolute metabolite levels A) and B) were calculated using linear mixed effect models with an interaction of time and treatment and a random effect of replicate. For N-carbamoyl-aspartate, a normal distribution of the residuals could not be assumed and therefore a non-parametric linear mixed effect model with an interaction of time and treatment and a random effect of replicate was performed. Afterwards, a contrast analysis was done to calculate the p-values between specific groups. *p < 0.05, **p < 0.01, ***p < 0.001. (TIF) [file ppat.1012036.s005.tif]

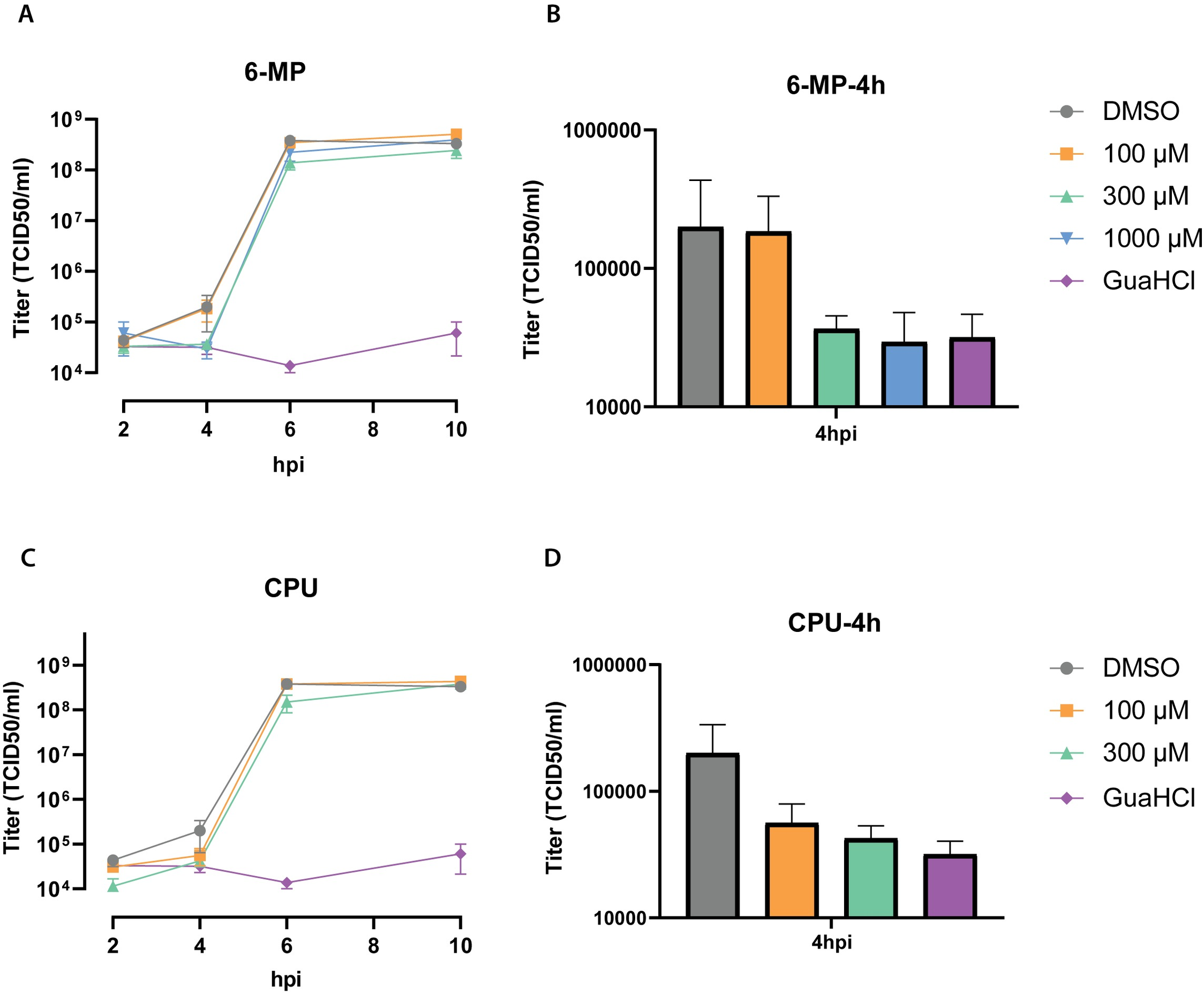

Supplement: S6 Fig — Growth kinetics of CVB3 in HeLa R19 cells in the presence of different concentrations of 6-MP (A, B) or CPU (C, D). The cells were infected, treated with the compounds directly after infection, lysed at 2, 4, 6, or 10 hpi and titrated on HeLa R19 cells to determine the TCID50/ml (MOI 5; mean ± SEM of triplicates; one experiment). Guanidine hydrochloride (GuaHCl) is a known replication inhibitor. A,C) Growth curve of all the included time points. B,D) Bar graph of the 4h time point. A two-way ANOVA was performed, but results are not significant. (TIF) [file ppat.1012036.s006.tif]

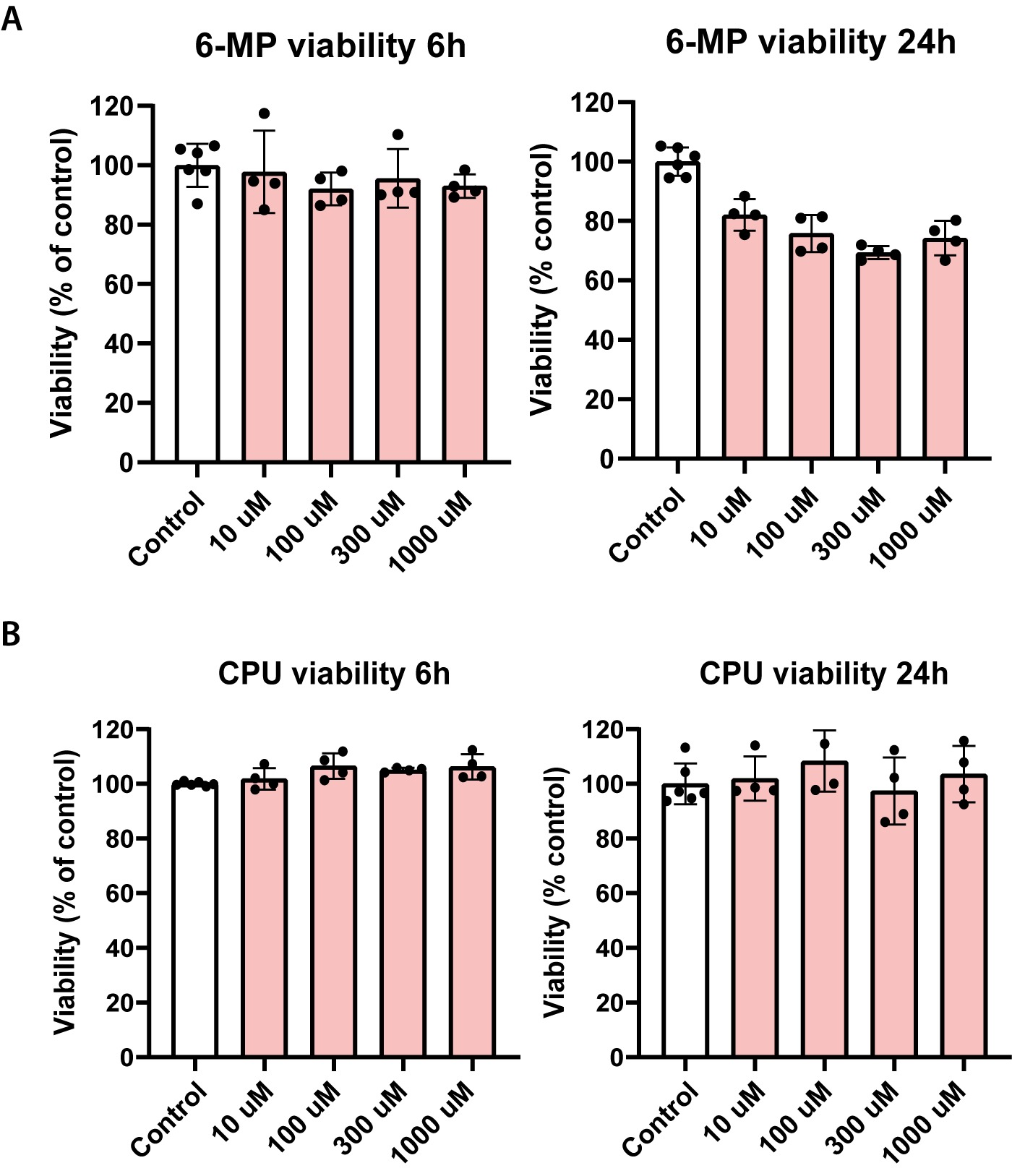

Supplement: S7 Fig — A) A representative MTS assay performed in parallel with the luciferase assay depicted in Fig 3A (mean ± SD). The cells were exposed to the different 6-MP concentrations for either 6h or 24h after which a MTS assay was used to determine the viability of the cells. B) A representative MTS assay performed in parallel with the luciferase assay depicted in Fig 3E (mean ± SD). The cells were exposed to the different CPU concentrations for either 6h or 24h after which a MTS assay was used to determine the viability of the cells. (TIF) [file ppat.1012036.s007.tif]

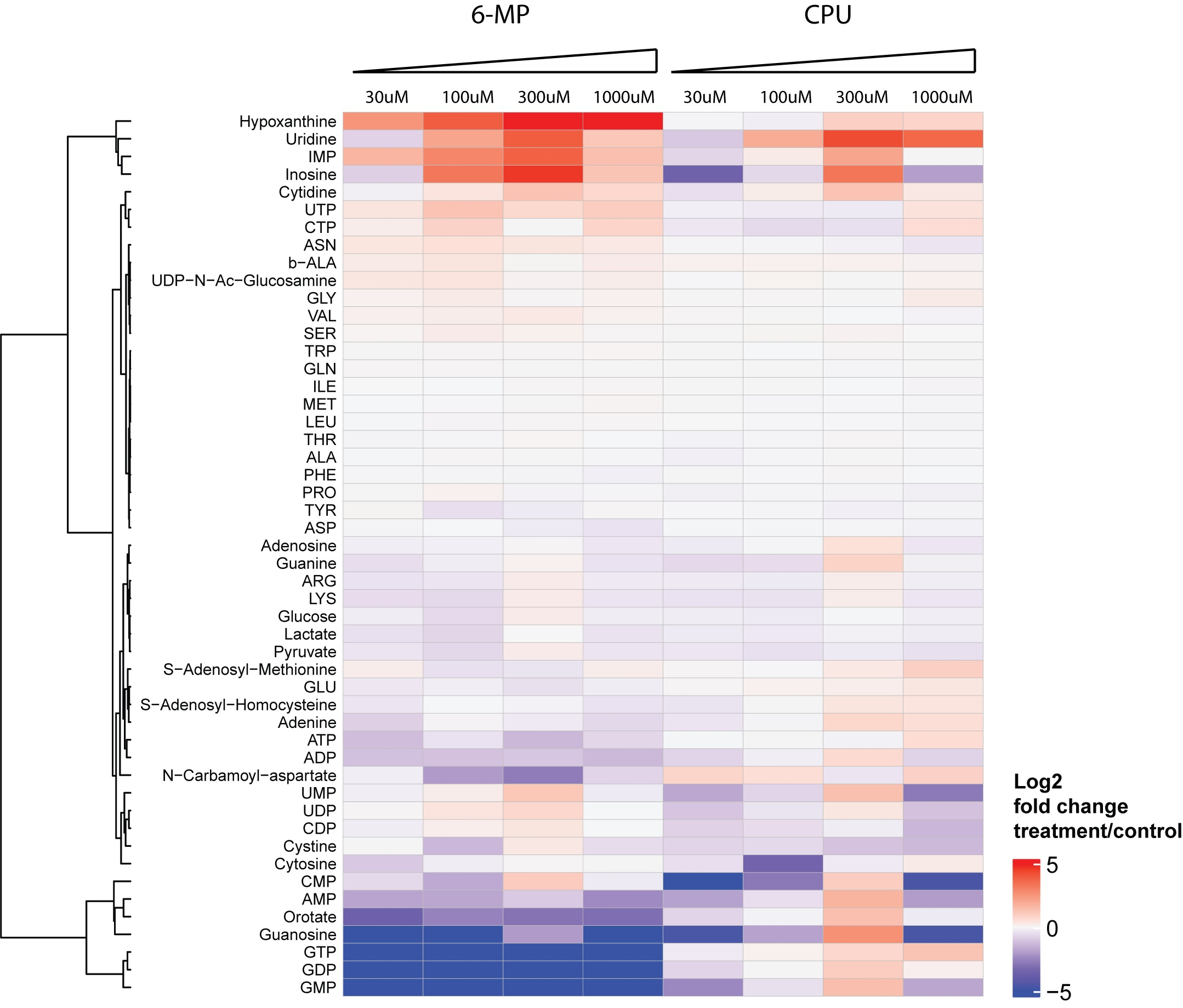

Supplement: S8 Fig — 13C-glucose isotope tracing study in control- and 6-MP or CPU treated HeLa R19 cells (three replicates; one experiment). Cells were exposed to different concentrations of 6-MP or CPU (30 μM, 100 μM, 300 μM, 1000 μM). The cells were lysed after at 6h and measured by LC-MS to identify metabolites and quantify the different isotopologues. The different isotopologues are not distinguished in this Figure. Heatmap showing log2 fold changes of intracellular metabolites compared to control-treated cells. (TIF) [file ppat.1012036.s008.tif]

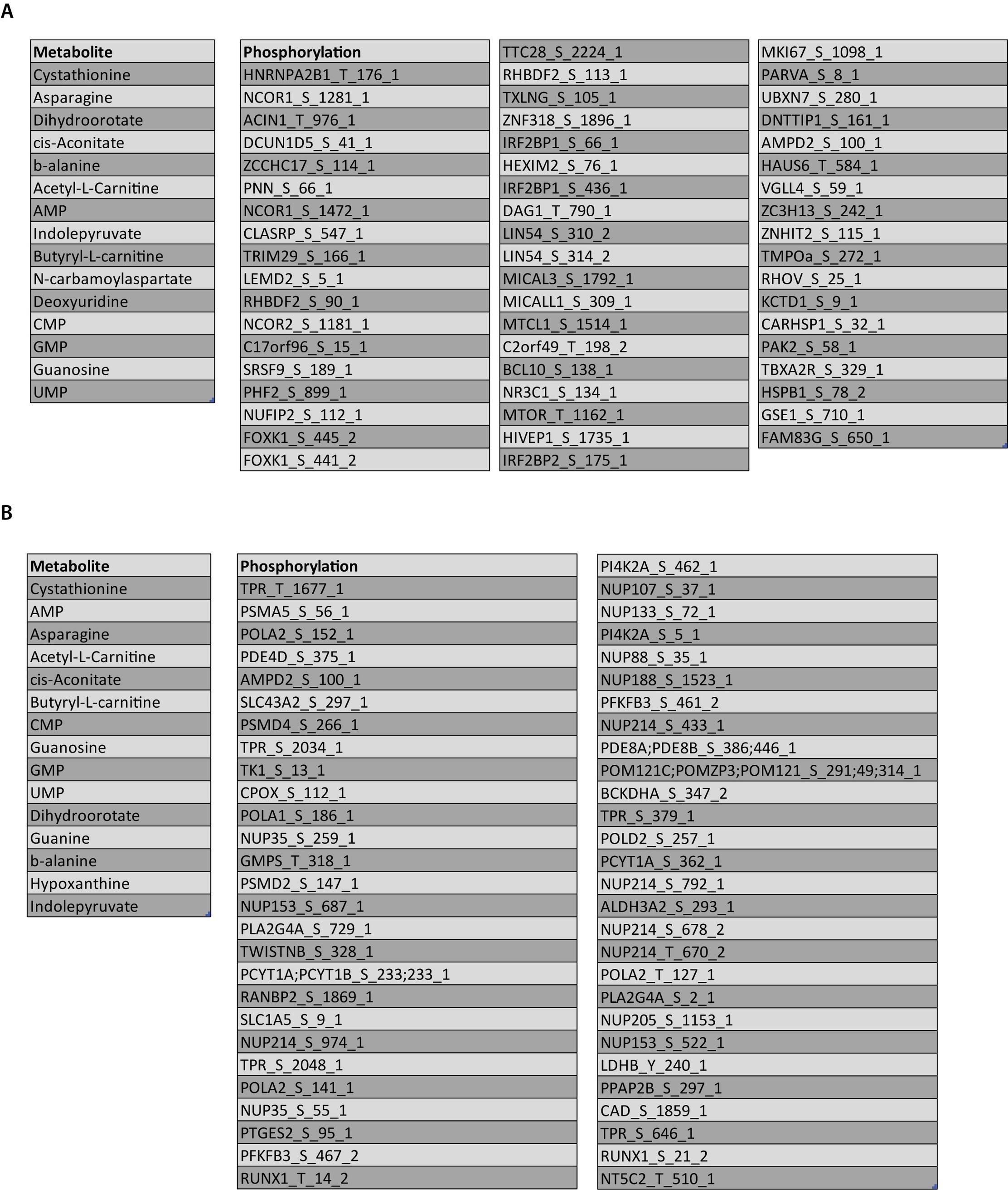

Supplement: S9 Fig — A) Extracted metabolites and phosphorylations of the DIABLO analysis (supervised analysis of multiple omics datasets) of the metabolomics dataset (MOI 5) and the total phosphoproteomic dataset (MOI 10) of mock and CVB3 infected HeLa R19 cells. The phosphorylations contain the protein, the phosphorylated site and whether this site is quantified on a singly (_1) or doubly (_2) phosphorylated peptide. B) Extracted metabolites and phosphorylations of the DIABLO analysis (supervised analysis of multiple omics datasets) of the metabolomics dataset (MOI 5) and the phosphoproteomic dataset filtered for metabolic proteins (MOI 10) of mock and CVB3 infected HeLa R19 cells. The phosphorylations contain the protein, the phosphorylated site and whether this site is quantified on a singly (_1) or doubly (_2) phosphorylated peptide. (TIF) [file ppat.1012036.s009.tif]

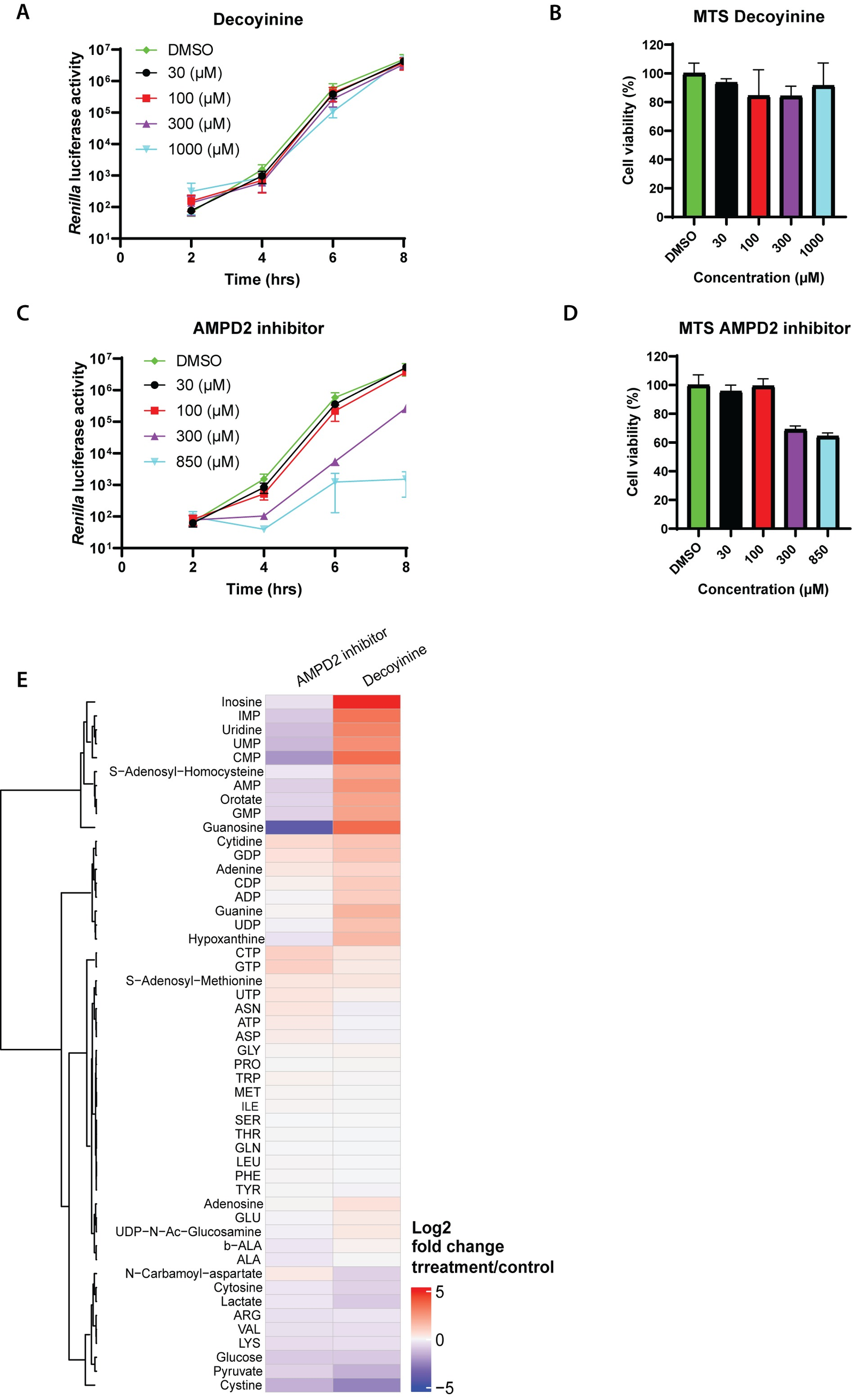

Supplement: S10 Fig — A CVB3 luciferase reporter virus carrying a Renilla luciferase (Rluc CVB3) was used to study the replication of CVB3 in the presence or absence of compounds inhibiting the salvage pathways. A, C) Luciferase levels in cells infected with Rluc CVB3 (MOI 0.1) in the presence of different concentrations of Decoyinine (A) or AMPD2 inhibitor (C). Cells were lysed at 2, 4, 6, 8 hpi. Representative data of three independent experiment are depicted (mean ± SD of 3 technical replicates). B,D) MTS assay performed in parallel with the luciferase assay depicted in A and C (mean ± SD). The cells were exposed to the different Decoyinine (B) or AMPD2 inhibitor (D) concentrations for either 8h after which a MTS assay was used to determine the viability of the cells. E) 13C-glucose isotope tracing study in control- and decoyinine (100 μM) or AMPD2 inhibitor (100 μM) treated HeLa R19 cells (three replicates; one independent experiment). The cells were lysed after 6h and measured by LC-MS to identify metabolites and quantify the different isotopologues. The different isotopologues are not distinguished in this Figure. Heatmap showing log2 fold changes of intracellular metabolites compared to control-treated cells. (TIF) [file ppat.1012036.s010.tif]

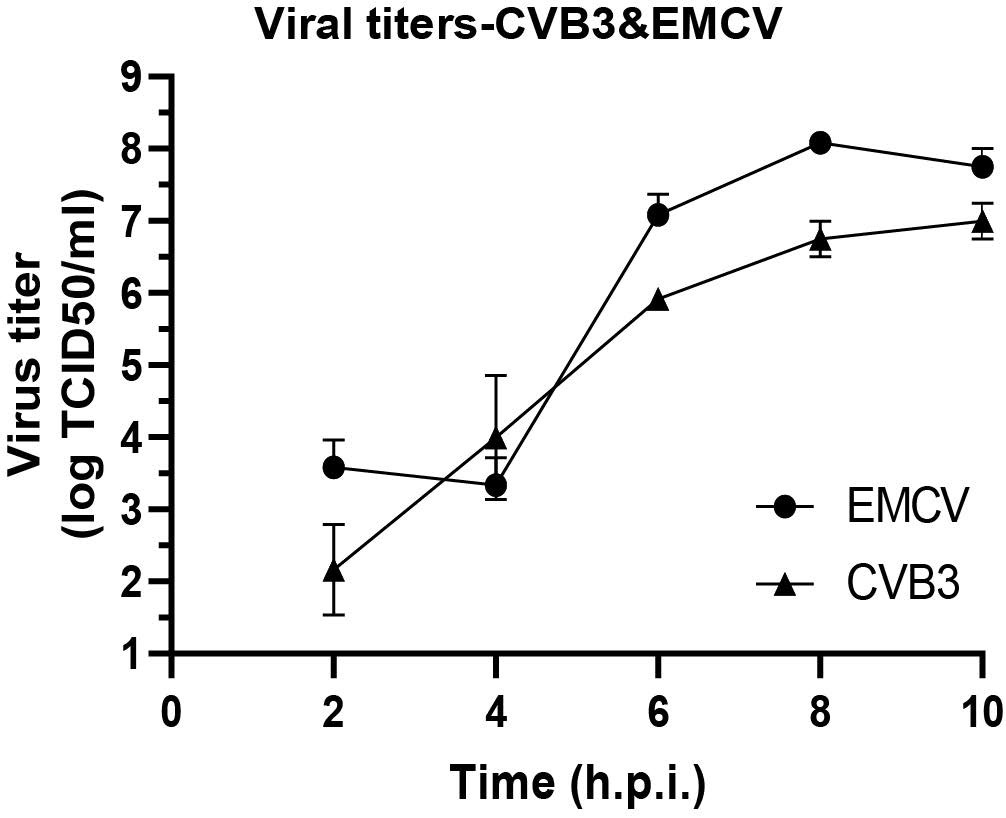

Supplement: S11 Fig — Growth kinetics of CVB3 and EMCV in HeLa R19 cells, titrated on HeLa R19 cells (MOI 2; mean and SD of triplicates; one experiment). The cells were infected, lysed at 2, 4, 6, 8 or 10 hpi and titrated to determine the TCID50/ml. (TIF) [file ppat.1012036.s011.tif]
