# Supplementary material for: Modulation of nucleotide metabolism by picornaviruses
Source: PLoS Pathog. 2024 Mar 8;20(3):e1012036. doi: 10.1371/journal.ppat.1012036 (PMC10923435; doi:10.1371/journal.ppat.1012036)
Supplement: Attachment — Submitted filename: response to reviewers.docx [file ppat.1012036.s013.docx]

Dear sir/madam,

We would like to thank the reviewers for their constructive input to improve the quality of our manuscript. Please find a point-by-point response to the comments.

In order to revise the manuscript according to the reviewers feedback, two new authors have been added. Marleen Zwaagstra performed and analyzed the experiment testing whether 6-MP and CPU had any effect on CVB3 virus production and Chris van de Lest was involved in the statistical analysis of the metabolomics data in the manuscript. Because their help was needed to finalize the manuscript, they have been added as authors. All authors have agreed to this and signed a form specifying the changes.

We hope the manuscript is now ready for publication.

Yours sincerely,

Celia Berkers

Reviewer's Responses to Questions

**Part I - Summary**
Please use this section to discuss strengths/weaknesses of study, novelty/significance, general execution and scholarship.

Reviewer #1: The authors conducted tracing experiments using [13C]-glucose to investigate the metabolome of infected cells at various time points throughout the infection. This approach allowed for a more accurate identification of the activities within metabolic pathways, which in turn provided a thorough comprehension of how the virus affects metabolic regulation. Additionally, the authors utilized previously acquired phosphoproteomic data from CVB3-infected cells to gain further insight into the underlying mechanisms that drive metabolic alterations during infection. The findings of this study provide compelling evidence that both CVB3 and EMCV infections enhance metabolite levels in the purine and pyrimidine pathways by promoting nucleic acid and nucleotide degradation, as well as facilitating nucleotide recycling, rather than through increased de novo synthesis of nucleotides.
This study is relevant because our understanding of how picornaviruses remodel cellular metabolism remains limited. Generally, viruses have the capability to actively reprogram glucose, glutamine, fatty acids, and nucleotides metabolism. This reprogramming not only facilitates the availability of necessary building blocks for viral replication but also acts in response to innate immune responses.
Interestingly, when a cell is infected with CVB3 at 4 hpi, the levels of nucleobases (such as uracil or hypoxanthine), nucleosides (such as uridine or guanosine), nucleotide monophosphates (like GMP, UMP), and nucleotide diphosphates (such as GDP, UDP) experience a significant increase. In contrast, intermediates of pyrimidine de novo synthesis show a pronounced increase during CVB3 infection. It would be good to provide folds of change to better represent the changes in nucleotide accumulation.

The heatmaps are composed of the (log2) foldchanges between CVB3 and Mock.

Reviewer #2: This manuscript reports the effect of picornavirus infection on nucleotide metabolism and its role in viral replication. The study is primarily based on carbon flux metabolomics and phosphoproteomics to make correlations between signal transduction via mTOR and changes in nucleotide metabolism in cells.

Reviewer #3: In this study the authors use a HELA cell line to study metabolic changes induced by picornaviruses, CVB3 and in one experiment, EMCV. For CVB3 they show metabolomic data indicating that infection increases pools of nucleobases, nucleosides, NMPs and NDPs but interestingly not NTPs. They also find strong decreases in pyrimidine synthesis intermediates which indicates that de novo nucleotide synthesis may be inhibited. They also use uniformly labeled C-13 glucose tracing to show that after infection there is decreased higher level labeling (M >5) of nucleosides, NMPs and NDPs further indicating that there is likely inhibition of de novo purine and pyrimidine synthesis. They show that the levels of (M + 5) typically derived from the labeled ribose ring and the nucleotide salvage pathway do not strongly increase after 4 hours as it does in the uninfected cells. However, they claim multiple times that this indicates increased salvage pathway, but the data do not clearly show this. While it is possible due to increased overall purine and pyrimidine levels that the salvage pathway increases somewhat, they do not clearly show this. They also perform metabolomic and C-13 glucose tracing in HUH cells, a transformed liver line and see many similar results. However, they do not address that there is a major difference between cell lines and that there is an increase in NTPs as well as NMPs and NDPs. Drug inhibition of the purine or pyrimidine salvage pathway leads to modest inhibition of viral replication, though they don’t show production of infectious virus, only luciferase assays. They never explain the decrease in NTPs and the relationship to viral replication, though this could be relevant. They include earlier phosphor-proteomic data to show that a number of nucleotide synthesis and degradation pathway enzymes are differentially phosphorylated. However, none of these studies are backed up with viral replication data or significant evidence they are involved. Finally they show metabolomic data for ECMV and find many similarities in purine and pyrimidine metabolism to CVB3 infected HELA cells.

**Part II – Major Issues: Key Experiments Required for Acceptance**
Please use this section to detail the key new experiments or modifications of existing experiments that should be absolutely required to validate study conclusions.

Generally, there should be no more than 3 such required experiments or major modifications for a "Major Revision" recommendation. If more than 3 experiments are necessary to validate the study conclusions, then you are encouraged to recommend "Reject".

Reviewer #1:

*Given that [13C]-glucose is introduced to the culture simultaneously with the initiation of infection, one important point to clarify is the speed at which the incorporation of [13C] from de novo synthesis of nucleotides is expected to occur. This is particularly crucial considering the rapid replication of the virus. Are there any positive controls, especially during the early timepoints, to determine if labeling has not yet reached a steady-state? This would help assess the levels of nucleotides derived from both de novo and salvage pathways.*

The labeling of some of the nucleotide precursors such as ribulose-5P, is very fast, with labeling approaching steady-state within 2h after infection (=M+5, we added this figure as Fig. S2 to line 809-811 in the manuscript (figure legend)). This labeled ribose becomes part of nucleotides due to either *de novo* nucleotide synthesis or salvage. Labeling of the nucleobases is generally slower, depending on the cell line/cell type used. Since HeLa cells are cancers cells and divide fast, the labeling of nucleotides is relatively fast as well. Although the synthesis of nucleotides is fast in HeLa cells, not all nucleotides are completely labeled within our time frame (both ribose and nucleobase labeled) and our data suggest (Fig. 2C and a 10h time point that we did not include in our final analysis)) that steady-state labeling is not yet reached within 8 hours. Our 10h time point was not included in our final analysis, because virus induced cell death occurs after 8 hours which could affect the results. However, because we compare infected cells with mock-treated cells at every time point, we can conclude that there is less *de novo* nucleotide synthesis in infected cells compared to the mock and that this is not merely a delay (over the course of infection, the labeling does not increase steadily as is the case in the mock). Importantly, the reviewer does endorse our conclusions: “*However, the authors support their conclusions by reporting the increasing levels of nucleotide monophosphate, nucleosides, and nucleobases from 6 hours onwards. Additionally, it is noted a decrease in nucleotide diphosphates and triphosphates after 6 hours. This is indeed consistent with their hypothesis.”*

We clarified this question in line 144-148 of the manuscript.

*Then, they proceeded to investigate the potential impact of a pharmacological intervention on the savage pathway. Utilizing a replicon system, they explored the effects of inhibiting the purine salvage pathway. Specifically, they employed 6-mercaptopurine, a compound known to target HGPRT, an indispensable enzyme within the purine salvage pathway. At 4 hours post-infection (hpi), they observed a slight decline in RNA synthesis, equivalent to a reduction of roughly 5 to 8-fold in RNA accumulation. One issue with this result is that the inhibitor may have some pleiotropic effect resulting in reduction of virus replication. Is there any way to address this concern?*

This is a very relevant question, since a lot of compounds do have off target effects. We used both 6-MP and CPU to study the salvage pathway during CVB3 infection. We used both compounds in a metabolomics study to address whether indeed these compounds would affect nucleotide metabolism and no other metabolic pathways (supplementary S6). In this analysis we find that the main effects of these compounds are on the levels of nucleotides and nucleobases. Besides inhibiting purine salvage, 6-MP is also known to inhibit *de novo* nucleotide synthesis of purines. However, CVB3 already inhibits *de novo* nucleotide synthesis itself and therefore this effect of 6-MP is likely to be less important than inhibiting the salvage pathway. Although we performed experiments to study off-target effects of 6-MP on metabolism, we cannot exclude that 6-MP has off-target effects outside of metabolism that could influence CVB3 replication. All in all, we believe that in our system 6-MP mainly affects nucleotide metabolism in control cells and mainly nucleotide salvage during CVB3 infection. We clarified this in line 197-200 of the manuscript.

*The authors then proceeded by integrating the metabolomics dataset with the phosphoproteomic data of CVB3-infected HeLa R19 cells. Through this intriguing analysis, they discovered five proteins implicated in nucleotide metabolism. Collectively, the integration of phosphoproteomic and metabolomic data suggests that nucleotide metabolism experiences alterations not only at the metabolome level but also at the phosphoproteome level. Inhibition of one of the enzymes discovered, AMPD2, had only a modest effect when inhibiting on CVB3 replication. This suggests that these enzymes are not essential for CVB3 replication.
This fails to provide a clear mechanistic understanding of how CVB3 and EMCV However, I am of the opinion that the study has been meticulously executed, with the discussion of the outcomes and conclusions lucidly articulated. The significance of this study lies in its ability to illuminate a crucial yet poorly understood facet of RNA virus replication.*

With the combined analysis of the metabolomic and phosphoproteomic data we show that nucleotide metabolism is altered at different levels. We do not only observe changes with metabolomics, but also with phosphoproteomics. Of course, we would like to understand mechanistically how this is interconnected, but this is very challenging. For multiple phosphorylations (including those on GMPS and AMPD2) we do not know their exact function. The fact that the inhibitors against these enzymes do not/or only modestly affect CVB3 replication means that the activation of these enzymes is likely not essential. However, it could be that the changed phosphorylation on these enzymes already inhibits the function of the enzyme (and therefore we do not observe drastic effects). Another possibility is that compensation and/or redundancy in the affected pathways masks effects of inhibitors of these enzymes. This might especially be true for AMPD2, since this enzyme is not a rate-limiting enzyme. So, although studying the mechanisms of the phosphorylations on the nucleotide regulating enzymes proves to be challenging, we do show that nucleotide metabolism is modified on both the metabolite and phosphorylation levels. This indeed illuminates a crucial yet poorly understood facet of RNA virus replication. We clarified this in line 300 to line 301 of the manuscript.

Reviewer #2:

The results are new and interesting, and the manuscript is well written. 
*1. There is no statistical analysis on Figs 1,2, 3 and 4. There is no indication of the type of statistical analysis used for the data in Fig 3.*

Thank you for this question. We included statistics in figure 1,2,3,5, S3,S4,S5 and described it in line 119-123, 179-183, 385-389, 576-588, 817-822, 826-830,836-841 of the manuscript. We now have statistics in fig 1-5 and S3-S5.

In figure 3, we do indicate the statistical method used for 3C and 3G: ‘ Statistical analysis was done using an ANOVA. For 6-MP, not every concentration was included in all seven independent experiments, but every concentration is measured in at least three independent experiments with three replicates. ***p < 0.001, ****p < 0.0001.’

In figure 4, the analysis is explained in the legend of the figure as well as in the Material & Methods section of the paper. This method does not give p values, because that is not the goal of this method. DIABLO, part of the MixOmics R package, is an explorative method to find connections between different datasets.

We noticed we forgot to include the type of statistical analysis that was performed for figure 4D. We now included this to line 324 of the manuscript.

*2. Phosphorylation of proteins must be confirmed by WB*

The phosphoproteins that came out of the analysis have been quantified using mass spectrometry, which is a very sensitive method to study protein abundance. Validation provides additional confirmation, but unfortunately, for the phosphoproteins that we continued studying using compounds (AMPD2 S100, GMPS T318), there are no WB antibodies available.

*3. The role of mTOR signaling must be confirmed by its pharmacological or genetic inhibition.*

We thank the reviewer for the suggestion. Earlier work of our lab revealed that mTORC1 is inhibited during CVB3 infection (both phosphoproteomic and WB analysis)[1]. Pharmacologic or genetic inhibition of mTORC1 will therefore not be useful to study the role of mTORC1 in CVB3 infection. As CAD, a downstream target of mTORC1 and involved in *de novo* nucleotide pyrimidine synthesis, is also inhibited by the inhibition of mTORC1, inhibiting CAD is also irrelevant.

[1] Giansanti P, Strating JRPM, Defourny KAY, Cesonyte I, Bottino AMS, Post H, et al. Dynamic remodelling of the human host cell proteome and phosphoproteome upon enterovirus infection. Nat Commun. 2020 Aug 28;11(1):4332. Available from: http://dx.doi.org/10.1038/s41467-020-18168-3

*4. Does inhibition of nucleotide salvage reduces viral protein synthesis and progeny release?*

Thank you for this question, we appreciate the suggestions. As per the reviewer’s suggestion, we tested the effect of nucleotide salvage inhibition on virus production and progeny release and included the data as fig S6 to line 843-848 (figure legend) and line 194-195 of the manuscript.

Reviewer #3:

*1. The metabolomics and tracing data are mostly performed in uninfected and infected HELA cells. HELA cells express high levels of Human papillomavirus E6 and E7 which are known to alter cellular metabolism and would mask many changes in metabolism that might be induced by picornaviruses.*

This is a very relevant comment. Indeed, cancer cells are metabolically different from primary cells. And as you indicate, this changed metabolism might mask effects of CVB3 on metabolism that we could observe in more primary cells. HeLa cells are widely used in the picornavirus field and that is why we performed the bulk of our studies in these cells. We are exploring options to use primary cells and/or stem cell derived cells, but these studies are still in the early phase and will be the topic of a future manuscript.

*2. They do not address why nucleobases, nucleosides, NMPs and NDPs are induced by infection but not NTPs in HELA cells. There is also limited discussion on the differences seen in HUH cells, namely that NTPs are upregulated only in HUH cells.*

We appreciate the question. In line 155-158 of the manuscript we describe the following:

*“Whereas the levels of nucleotide monophosphate, nucleosides and nucleobases are increased from 6 hours onwards, the levels of nucleotide diphosphates and triphosphates decrease after 6 hours, suggesting degradation of nucleotide di- and triphosphates to nucleotide monophosphates, nucleosides and nucleobases.”*

We believe that apart from degradation of nucleic acids, there is also degradation of the nucleotides themselves, which leads to an increase of nucleobases, nucleosides, NMPs (and at 6hpi also NDPs), while at 8 hpi NDP and NTP decrease. Another likely factor contributing to the alterations in nucleotide levels is replication, where the NTPs are used to create more viral genomes.

We acknowledge that there are differences in cell lines in terms of both levels of nucleotides and pathways used. What we consistently observe is that metabolites in the nucleotide pathways generally increase and that the main increase is coming from unlabeled nucleotides and the M+5 fraction. We also observe that the levels of N-carbamoyl-aspartate (one of the intermediates) of the *de novo* nucleotide synthesis is decreasing over time compared to the mock, suggesting that also in the Huh7 cells nucleotide metabolism is modulated by CVB3 infection. We addressed this question by changing line 163-167.

*3. The salvage pathway does not appear to be strongly induced and if anything is flat or limited in the infected cells as compared to the uninfected cells as determined by the percentages of M+5 labeling. They state throughout that the salvage pathway is activated by infection which is not so clear in the data presented. They show inhibition of purine or pyrimidine salvage pathway has modest effects on replication which goes along with the limited activation. Additionally, they do not show production of virus, just increase in luciferase showing increased RNA replication of the virus. Importantly, they only show this modest effect at 4 hours post infection while the differences in purine and pyrimidine metabolism is mostly seen only at later times post infection.*

In our analysis, we mainly focus on the M+5 fraction to determine the activity of the salvage pathway. However, the M+5 fraction includes both salvage and *de novo* nucleotide synthesis, while M>5 is only *de novo* synthesis. During CVB3 infection, the M>5 fractions almost disappear, whereas the M+5 fraction decreases slightly compared to the mock but remains relatively stable over time. Together, the data suggest that during infection, the cell and virus switches from producing nucleotides from *de* novo nucleotide synthesis, degradation of nucleic acids and salvage to mainly producing nucleotides from degradation of nucleic acids and salvage. Since it is difficult to separate salvage and *de novo* nucleotide synthesis in a black and white manner, that is why we describe our observation as ‘remains active’ and not ‘activated’ in line 159. In line 186 we changed ‘activated’ to ‘remains active’.

*4. While the combination of metabolomics and phosphoproteomics is potentially interesting, all of the phosphosite analysis is correlative and no effects of phosphorylation on purine or pyrimidine metabolism is shown in the infected cells. Also may of the phosphosites they discuss have unknown effects on the protein function so does not make a clear point. Furthermore, knockdown of the two of the enzymes that have altered phosphorylation on specific sites, has no effect on viral replication indicating that they are likely not relevant to the changes in infected purine and pyrimidine metabolism.*

We address this question in question 3 of reviewer 1.

5. They draw many large conclusions that they do not go far enough experimentally to demonstrate in the manuscript
a. They state “We present evidence that both CVB3, an enterovirus, and EMCV, a cardiovirus, increase purine and pyrimidine nucleotide levels by promoting degradation of nucleic acids and nucleotides as well as triggering salvage pathways.” However, they do not show direct evidence that the degradation is activated and the data for the salvage pathway labeling is not as clear as they indicate as it appears to level off faster than in the uninfected cells indicating it is partially shut down not activated.

During our metabolomics experiments we use 13C glucose to trace/label which pathways are active and which are inhibited. Metabolites that are unlabeled are already present in the cell before the labeling started (nucleotides that were already produced prior to labeling and infection), are produced from unlabeled intermediates (ribose or nucleobase that was already produced) or are ‘released’ from degradation of upstream metabolites/entities (RNA/DNA). We assume that the amount of metabolites that is unlabeled prior to labeling and infection is the same between the mock and CVB3-infected cells. In this case, the likely explanation of a rise in unlabeled nucleotides is degradation of nucleic acids. Our conclusion of the salvage pathway was explained in comment/question number 3. We have adjusted ‘triggering salvage pathways’ to ‘sustaining salvage pathway activity’ in line 401 of the manuscript.

b. IN the discussion section, they extensively discuss RNA degradation pathways and their potential relevance to the increased purines and pyrimidines. However, they focus on enzymes like RNaseL and XRN/AUF, that are inhibited by picornaviruses which does not make sense for their hypothesis that RNA degradation is increased.

This is a very good question. The main focus and dogma of the literature concerning RNA degradation by picornaviruses and other RNA viruses is that viruses modulate/inhibit RNA degradation to prevent the degradation of their own genome. However, our data suggests that the degradation of RNA might increase the nucleotide pool that can be used to produce more viral RNA. However, the interaction of RNA decay, genome decay and genome production needs to be studied in more detail to better understand this process. We now clarified this in the discussion in line 461-462 and line 473-476.

**Part III – Minor Issues: Editorial and Data Presentation Modifications**
Please use this section for editorial suggestions as well as relatively minor modifications of existing data that would enhance clarity.

Reviewer #1: (No Response)

Reviewer #2: (No Response)

Reviewer #3: (No Response)
